# Challenging the notion of Aedes aegypti as the primary chikungunya virus vector: insights from Kédougou, Southeastern Senegal

**DOI:** 10.21203/rs.3.rs-6865029/v1

**Published:** 2025-07-01

**Authors:** Alioune Gaye, Moussa Moïse Diagne, Diawo Diallo, El Hadji Ndiaye, Marie Henriette Dior Ndione, Moussa Gaye, Idrissa Dieng, Madeleine Dieng, Mouhamed Kane, Safietou Sankhe, Babacar Diouf, Faty Amadou Sy, Caroline Weldon, Ibrahima Dia, Scott C. Weaver, Mawlouth Diallo

**Affiliations:** Institut Pasteur de Dakar; Institut Pasteur de Dakar; Institut Pasteur de Dakar; Institut Pasteur de Dakar; Institut Pasteur de Dakar; Institut Pasteur de Dakar; Institut Pasteur de Dakar; Institut Pasteur de Dakar; Institut Pasteur de Dakar; Institut Pasteur de Dakar; Institut Pasteur de Dakar; Institut Pasteur de Dakar; University of Texas Medical Branch; Institut Pasteur de Dakar; University of Texas Medical Branch; Institut Pasteur de Dakar

**Keywords:** Chikungunya outbreak, epidemic chikungunya vector, wild Aedes vectors, chikungunya amplification, Aedes furcifer

## Abstract

**Background:**

Chikungunya fever (CHIK) caused by the mosquito-borne chikungunya virus (CHIKV) and transmitted by *Aedes* mosquitoes, remains a public health burden throughout the tropics. During the CHIK outbreak in the southeastern Senegal in August 2023, an entomologic investigation was conducted to identify the vector(s) and characterize the virus strains.

**Methods:**

Adult mosquitoes were collected indoors and outdoors from houses of confirmed CHIK cases and their immediate neighborhoods using Prokopack aspirators and double-net traps and all water containers were inspected for aquatic stages. Mosquito pools were tested for CHIKV by RT-qPCR and positive samples were subjected to whole genome sequencing using Illumina iSeq system.

**Results:**

Animal watering points; bricks and tree holes were the most common sites for *Aedes aegypti* larvae and pupae. While immature *Ae. Aegypti* were found in all affected villages, with Breteau and Container indices exceeded the WHO epidemic thresholds, *Ae. furcifer* emerged as the most abundant host-seeking species in domestic areas. CHIKV was detected in 31 mosquito pools, primarily in *Ae. furcifer* (22 pools) and only one pool of *Ae. aegypti*. Other *Aedes* species accounted for 8 positive pools and *Anopheles gambiae*, the primary malaria vector, one pool. Phylogenetic analysis confirmed the close relationship between 2023 CHIKV strains circulating in humans and mosquitoes, and those responsible for the 2015 outbreak.

**Conclusions:**

Our study highlights the urgent need to include sylvatic mosquitoes in surveillance and control programs that until now have mainly focused on *Ae. aegypti*. Moreover, the potential role of *Anopheles gambiae* in the CHIKV transmission in Senegal warrants further investigation.

## Background

Chikungunya virus (CHIKV) is a mosquito-borne alphavirus of the family *Togaviridae*, that causes a febrile illness, often accompanied by severe joint pain as well as other common signs and symptoms including muscle pain, headache, nausea, fatigue and rash [1]. The joint pain associated with chikungunya can be debilitating and may persist for weeks or months [2]. Known to be endemic for at least decades in Africa and Asia [3], CHIKV has spread in recent years to other parts of the world, including Europe [4] and the Americas [5]. CHIKV is transmitted to humans by mosquitoes, with *Aedes aegypti* recognized as the principal epidemic vector in most locations worldwide, and *Ae. albopictus* as a secondary vector. In Africa sylvatic *Aedes* mosquitoes are considered to play an important role only in CHIKV maintenance in the enzootic cycle involving non-human primates living in the forest canopies [6, 7].

In West Africa, sylvatic CHIK amplification tends to occur cyclically, approximately every 4 years [8]. As reported in previous publications, an outbreak was detected in 2006 in southeastern Senegal with only 6 confirmed cases [9], coinciding with an outbreak in Nigeria and Cameroon [10, 11]. Subsequently in 2009, another outbreak affected Ghana, Ivory Coast and Burkina Faso, as well as southeastern Senegal, where 20 human cases were confirmed [12, 13]. More recently in 2015, another CHIK outbreak occurred in the Kedougou region of Senegal with 40 confirmed cases reported across its three administrative subdivisions: Kedougou, Salemata and Saraya [14].

Various factors are suspected to influence the cyclic emergence of CHIKV including climatic conditions such as temperature, rainfall and humidity, which impact the vectorial capacity of mosquitoes. Additionally, the herd immunity acquired during previous outbreaks may temporarily reduce the transmission intensity. Over time, CHIKV undergoes genetic changes that can affect its ability to replicate, be transmitted, or evade the host immune response. Moreover, human activities and movement can contribute to virus spread and its introduction into new areas with young, susceptible populations.

In August 2023, Senegal experienced a CHIK outbreak, with over 200 confirmed cases reported in the Kedougou region [15]. An entomological investigation was undertaken with the objective to identify the mosquito vectors involved and genetically characterize the virus responsible of the epidemic.

## Materials and Methods

### Study site

**Figure 1:**
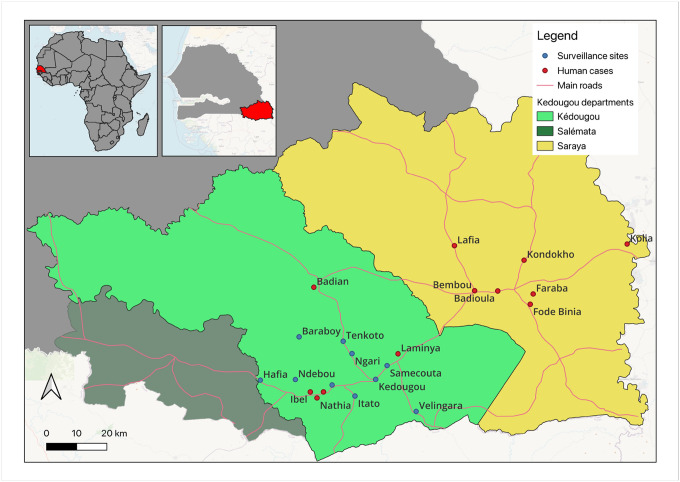
Mosquito sampling sites

Mosquito collections were carried out during August to September, 2023, across ten geographic sites in southeastern Senegal, encompassing five predominant land cover classes: village, agriculture, barren, savanna, forest as previously described [6]. In addition, 13 villages (Laminya, Nathia, Boundoucondi, Ibel, Badian, Fodé Binia, Lafia, Bembou, Faraba, Badioula, Kolia and Kondokho) where human chikungunya were confirmed, were investigated. Figure 1 shows villages with human cases (in red) and surveillance sites (in blue) where mosquitoes were sampled, Southeastern Senegal, August 2023. Adult mosquitoes were sampled across all biotopes and localities, while immature stages were sampled exclusively in villages where human cases were detected.

### Mosquito sampling

*Immature stages* sampling was performed indoor and outdoor of randomly selected human habitations. All artificial and natural water-holding containers were inspected, using a flashlight if necessary, and considered as positive when harboring at least one larva or pupa of *Ae. aegypti*. From each positive container, a sample of larvae and/or pupae was collected, reared to adulthood, and identified morphologically.

*Resting and host-seeking adult mosquitoes* were collected indoors and outdoors in each locality using a Prokopack aspirator [16]. Additionally, host-seeking mosquitoes were collected in the other landcover classes by aspiration in a double net baited by humans.

#### Sample processing.

Immature mosquitoes sampled from water container were reared to adulthood, while adults collected in nature were processed directly. Morphological identification of mosquitoes was performed on a chill table using appropriate keys [17–21]. Mosquitoes were pooled by species, sex, physiological status (engorged or unengorged), collection method, location, and date. Pools were then tested in the field laboratory for virus detection. Field-based Next Generation sequencing was also used for whole genome sequencing of positive samples.

#### Molecular detection.

Mosquito pools were homogenized in 1.5-ml Eppendorf tubes containing 500 μl of L-15 medium (Gibco BRL, Grand Island, NY, USA) using sterile pestles in a biosafety level 2 laboratory. The homogenates were centrifuged at 8,000 rpm for 10 min at 4°C. The supernatant was retained for further analyses, while mosquito debris was discarded.

For CHIKV detection, RNA was extracted from 140 μl of supernatant using the QiaAmp Viral RNA Extraction Kit (Qiagen, Heiden, Germany) following the manufacturer’s protocol. RNA was amplified using a one-step real-time RT-qPCR assay with the TIB Molbiol LightMix^®^ (Berlin, Germany). The 20 μL reaction volume consisted of 5 μL of extracted RNA, 2x Master Mix, 10 μM of specific primers (Forward: AAg CTY CgC gTC CTT TAC CAAg, Reverse: CCA AAT TgT CCY ggT CTT CCT) and probe (6FAM/CCA Atg TCY TCM gCC Tgg ACA CCTTT/TMR) targeting CHIKV in singleplex.

#### Whole Genome Sequencing and phylogenetic analysis.

Confirmed CHIKV-positive samples were processed for field-based Next Generation sequencing using the Twist Biosciences Comprehensive Viral Research Panel (CVRP) to obtain the whole viral genome. Enriched sample libraries were loaded onto an Illumina iSeq 100 sequencing system, and genome assembly was performed using the Chan Zuckerberg ID (CZ-ID; formerly IDSeq) platform [22].

All generated sequences were aligned using MAFFT with a representative CHIKV dataset covering the West African (WA), East-Central-South African (ECSA) and Asian genotypes. Maximum likelihood trees were generated using IQ-TREE [23] with a 1,000 bootstrap iterations, utilizing the best-fit substitution model determined by ModelFinder and 1,000 bootstrap replicates for statistical reliability and visualization was made with FigTree V1.4.4 [24].

### Data analysis

Data from larval surveys were used to estimate three key entomological indices: the Breteau Index (BI) defined as the number of containers positive for immature stages of *Ae. aegypti* per 100 housing units [25], the Container Index (CI) representing the number of containers positive for immature *Ae. aegypti* per 100 inspected water containers [26] and the Breeding Preference (BP) defined as the ratio of the percentage of positive containers (Y) to the percentage of that type of inspected container (X). The highest Y/X ratio indicated the preferred type of breeding site for mosquitoes. Epidemic thresholds for BI and CI were set at 5% and 3%, respectively, based on WHO standards [27]. The minimum field infection rates for CHIKV were calculated, including 95% confidence intervals (lower and upper limits), using the PooledInfRate software, version 4.0 [28]. Statistical differences were determined with a significance level set at p < 0.05.

## Results

### Collection of immature stages and adult mosquitoes.

A total of 742 water containers was inspected across the surveyed sites, showing significant variations in *Ae. aegypti* breeding preferences between administrative departments. In Saraya tree holes emerged as the most preferred sites, followed by bricks. In contrast, in Kédougou department, animal watering troughs and bricks were the sites most commonly inhabited by mosquito immatures (Table 1). At the village level, animal watering troughs were identified as the most commonly occupied containers in 9 out of 12 villages. Other notable immature habitats included tree-holes, bricks, water storage containers and tires. In all the villages investigated, the epidemic risk indices consistently exceeded the thresholds defined by the WHO across all surveyed villages (see additional file).

A total of 6209 adult mosquitoes belonging to 49 species and 8 genera were collected during the period of the CHIK outbreak. Of these, 31 mosquito pools comprising 8 species (7 *Aedes* and 1 *Anopheles* species) were found infected by CHIKV (Table 2). *Ae. furcifer* was the only species found infected across all biotopes. In the forest biotope, *Ae. taylori* was the most frequently infected species (p = 0.03, statistically significantly higher than *Ae. vittatus*,) followed by *Ae. furcifer*. In the savanna, *Ae. dalzieli* showed the highest infection rate (p = 0.02, statistically significantly higher than *Ae. vittatus*,) followed by *Ae. furcifer*. In village settings, *An. gambiae* was the most frequently infected species followed by *Ae. furcifer*, while in Barren and Agriculture environments, *Ae. furcifer* was the only infected species.

### Phylogenetic analysis

The phylogenetic analysis revealed that the 2023 CHIKV outbreak strain in Kedougou belongs to the WA genotype, forming a highly supported monophyletic group (bootstrap ≥ 95) with strains from the 2015 and 2005 outbreaks in the same region [29]. These sequences share 98.80–98.96% nucleotide identity with the reference strain HM045817 (2005), underscoring their close genetic relationship. Furthermore, the clustering of mosquito and human sequences confirmed that the same viral strain circulated between these hosts during the outbreak.

**Figure 2:**
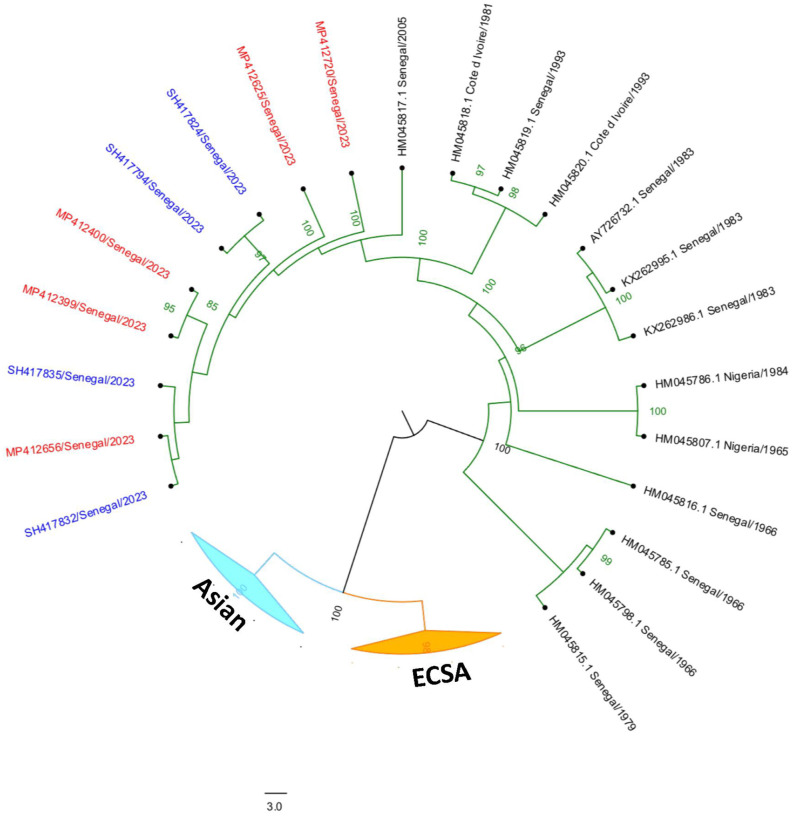
CHIKV sequences obtained from mosquitoes and humans during 2023 outbreak in southeastern Senegal Phylogenetic analysis of CHIKY sequences obtained from mosquitoes (labeled with red identifier numbers) and humans (labeled with blue identifier numbers) during the 2023 outbreak in southeastern Senegal. The sequences are grouped according to their genetic lineage, including those belonging to the West African, East/Central/South African (ECSA), and Asian genotypes.

Figure 2 provides results of the phylogenetic analysis of CHIKV sequences obtained from mosquitoes (labeled with red identifier numbers) and humans (labeled with blue identifier numbers) during the 2023 outbreak in southeastern Senegal. The sequences are grouped according to their genetic lineage, including those belonging to the West African, East/Central/South African (ECSA), and Asian genotypes.

## Discussion

Nearly worldwide, *Aedes aegypti* along with *Ae. albopictus* is considered the primary epidemic vector of several common, human-amplified arboviruses such as dengue, yellow fever, Zika and CHIKV [6, 30–33]. In fact, *Ae. aegypti* has been several times found associated with CHIKV in central [34], southern [35] and eastern parts of Africa [36], and also identified elsewhere in Africa as the main epidemic vector [37, 38]. Furthermore, populations of *Ae. aegypti* from South Africa [39], Cameroon [40], Senegal and Cape Verde [41, 42] have been shown to be experimentally competent for CHIKV transmission. Moreover, vertical transmission of CHIKV in *Ae. aegypti* has been demonstrated experimentally in India [37]. All of these results support a major CHIKV vector role for *Ae. aegypti*.

For many decades, *Ae. aegypti formosus*, the ancestral, sylvatic form that evolved into the domesticated *Ae. aegypti aegypti*, was considered as the only subspecies present in southern Senegal [43]. However, recent studies have shown the presence of both subspecies in Kédougou department [44] with larval development sites found in both forest and, more recently, in domestic environments. These changing distribution patterns are likely linked to deforestation driven by agriculture, gold mining and other human activities, leading to the domestication of wild *Ae. aegypti* populations in response to urbanization. Such adaptation to the domestic environment has also been observed in Nigeria [45] and Gabon [46].

In our study, *Ae. aegypti* was abundant in immature stages in all potential aquatic sites and the epidemic risk indices were above the thresholds defined by the WHO [25, 26]. In other words, the indices were sufficiently high for this species to ensure CHIK transmission. Also, at the adult stage *Ae. aegypti* were found during our investigation in all affected localities. However, CHIKV infection in *Ae. aegypti* was surprisingly low, with only one positive pool, representing just 3.2% of positives. Therefore, the main epidemic vector worldwide apparently did not play a major role in CHIKV transmission to humans during this outbreak. The zoophilic tendency of *Ae. aegypti* generally observed in West Africa [47] may explain this finding. The *Ae. aegypti formosus* form is significantly more abundant in the Kedougou area. However, it has been previously shown to be less endophilic and anthropophilic than the *Ae. aegyptiaegypti* and thus less involved in human-amplified arbovirus transmission [48]. Therefore, *Ae. aegypti formosus* probably plays no major role in either maintenance of the sylvatic cycle or the spillover of CHIKV to humans in this region [6].

Our findings highlight a significant involvement of wild mosquitoes in CHIKV transmission during this epidemic, suggesting epizootic amplification of the sylvatic cycle. CHIKV was detected in 29 pools of wild *Aedes* mosquitoes, predominantly *Ae. furcifer* (22 pools), which accounted for 71% of positives. Historically, *Ae. furcifer* has been found most frequently infected with CHIKV [49, 50] and is competent to transmit the virus [51, 52].

The same profile of infected mosquitoes was previously observed in 2009–2010, when among the 42 CHIKV-positive pools, most were wild mosquitoes. Only one pool of *Ae. aegypti* was found positive [6]. The same trend was observed during the 2015 amplification with the sylvatic vectors recording 93.7% of the CHIKV detections and only 6.25% (2/32 pools) for *Ae. aegypti* [53]. These findings corroborated our results and support the conclusion that *Ae. aegypti* does not play a significant role in CHIKV outbreaks in the Kedougou region.

The higher number of human cases (over 200 persons) recorded among men and young people over 15 years of age [15] supports the hypothesis that transmission occurred mainly outside of human dwellings. Sustained domestic transmission would be expected to affect age groups, including children under 15 years of age, who generally stay at home and spend more time indoors. *Ae. furcifer*, known to have great ecological plasticity [6], was frequently found infected and biting humans in villages at high density in Senegal [6, 43, 54] and elsewhere in Africa [55], indicating its major role in CHIKV transmission.

We also found during our investigation one PCR-positive pool of male *An. gambiae*. Other species within the *Anopheles* genus such as *An. funestus, An. coustani* and *An. domicola* have been found naturally infected with CHIKV in the Kédougou region during a previous study in 2009–2010 [6]. Subsequently in 2015, 3 pools of female *An. gambia* ewere found positive for CHIKV in the same area during a co-amplification with YFV and ZIKV [53], suggesting sustained CHIKV transmission by *An. gambiae*. Vector competence studies of *An. gambiae* showed a CHIKV infection rate of 43% at day 7 post-bloodmeal, although viral titers in the mosquitoe hemocoel were low and CHIKV-positive saliva was not detected [56]. Moreover *An. gambiae* and *An. funestus*, both present in the Kédougou region and transmitting malaria parasites, are the only known vectors of o’nyong-nyong virus (ONNV), a close relative of CHIKV [57, 58]. Also, *An. stephensi* was recently shown to be a competent ONNV vector [59]. All of these findings suggested that malaria vectors should be monitored for their potential role in CHIKV transmission in Senegal and elsewhere in Africa.

Phylogenetic analysis confirmed that the same CHIKV strain circulated between humans and mosquitoes during 2023, harboring shared mutations compared to CHIKV strains that circulated in 2005 and 2015 [15]. These mutations deserve further study as CHIKV mutations can greatly affect vector host range [60].

## Conclusions

This study highlights the persistence of an endemic CHIKV strain in a sylvatic transmission cycle primarily, emphasizing the significant role of wild, sylvatic *Aedes* species in CHIKV amplification and transmission to humans. Our findings underscore the urgent need to assess the insecticide resistance status of these species and integrate them into vector control programs. Further studies are required to better understand the many factors that contribute to the amplifications of arboviruses like CHIKV at the interface of humans and both sylvatic and urban environments, which can facilitate virus re-emergence and transmission.

## Figures and Tables

**Table 1:** *Aedes aegypti* immature habitats at the department level, Kedougou region, August 2023

Departments	Container type	Containers inspected (X%) *	Positive containers (Y%) ^ψ^	Immature site Preference (Y%/X%)
SARAYA	Clay jars	85 (14.10)	6 (3.11)	0.22
	Water storage containers	152 (25.21)	11 (5.70)	0.23
	Tires	62 (10.28)	24 (12.44)	1.21
	**Bricks**	106 (17.58)	88 (45.60)	**2.59**
	Animal watering	13 (2.16)	9 (4.66)	2.16
	Abandoned containers	181 (30.02)	52 (26.94)	0.90
	Basins	2 (0.33)	1 (0.52)	1.56
	**Tree holes**	2 (0.33)	2 (1.04)	**3.12**
	TOTAL	603	193	
KEDOUGOU	Clay jars	32 (24.06)	4 (5.97)	0.25
	Water storage containers	26 (19.55)	5 (7.46)	0.38
	Tires	14 (10.53)	8 (11.94)	1.13
	**Bricks**	29 (21.80)	29 (43.28)	**1.99**
	**Animal watering**	6 (4.51)	6 (8.96)	**1.99**
	Abandoned containers	26 (19.55)	15 (22.39)	1.15
	TOTAL	133	67	

* X% = Containers inspected /Total examined

ψ Y% = Positive containers /Total Positives

**Table 2:** Infection rates of mosquito species associated with CHIKV in different biotopes, Kedougou, 2023.

Biotope	Species	Total Pos	Total mosquitoes	Total negative pools	Infection rate	Lower confidence	Upper confidence
Forest	*Ae. africanus*	1	179	85	5.586	0.001	0.031
Forest	*Ae. furcifer*	8	941	144	0.008	0.004	0.017
Forest	*Ae. luteocephalus*	1	190	85	0.005	0.001	0.029
Forest	*Ae. taylori*	3	129	30	0.023	0.008	0.066
Savanna	*Ae. dalzieli*	1	5	3	0.2	0.036	0.624
Savanna	*Ae. furcifer*	3	273	45	0.011	0.004	0.032
Savanna	*Ae. vittatus*	1	380	46	0.002	0.0005	0.015
Village	*Ae. aegypti*	1	149	65	0.007	0.001	0.037
Village	*Ae. furcifer*	7	508	70	0.014	0.007	0.028
Village	*An. gambiae*	1	68	22	0.015	0.003	0.079
Barren	*Ae. furcifer*	3	320	36	0.009	0.003	0.027
Agriculture	*Ae. furcifer*	1	259	41	0.004	0.001	0.022

## Abbreviations

Ae.: Aedes
An.: Anopheles
BI: Breteau index
BP: Breeding Preference
CHIK: Chikungunya fever
CHIKV: Chikungunya virus
CI: Container index
CVRP: Comprehensive Viral Research Panel
CZ-ID: Chan Zuckerberg ID
ECSA: East-Central-South African
MAFFT: Multiple Alignment using Fast Fourier Transform
NY: New York
ONNV: o’nyong-nyong virus
RNA: Ribonucleic Acid
RT-qPCR: Real time quantitative polymerase chain reaction
USA: United State of America
WA: West African
WHO: World Health Organization
YFV: Yellow fever virus
ZIKV: Zika virus
